# High-Sensitivity and Wide-Range Flexible Ionic Piezocapacitive Pressure Sensors with Porous Hemisphere Array Electrodes

**DOI:** 10.3390/s24020366

**Published:** 2024-01-08

**Authors:** Bang Wu, Weiguang Wu, Rui Ma, Haobing Chen, Yilin Zhao, Yunfan Li, Xiao Lei, Feng Liu

**Affiliations:** 1 School of Power and Mechanical Engineering, Wuhan University, Wuhan 430072, China; 2 Hubei Key Laboratory of Electronic Manufacturing and Packaging Integration (Wuhan University), Wuhan University, Wuhan 430072, China

**Keywords:** pressure sensor, ionic polymer film, porous hemispherical microstructure, high sensitivity, wide measurement range

## Abstract

The development of high-performance flexible pressure sensors with porous hierarchical microstructures is limited by the complex and time-consuming preparation processes of porous hierarchical microstructures. In this study, a simple modified heat curing process was first proposed to achieve one-step preparation of porous hemispherical microstructures on a polydimethylsiloxane (PDMS) substrate. In this process, a laser-prepared template was used to form surface microstructures on PDMS film. Meanwhile, the thermal decomposition of glucose monohydrate additive during heat curing of PDMS led to the formation of porous structures within PDMS film. Further, based on the obtained PDMS/CNTs electrodes with porous hemisphere array and ionic polymer dielectric layers, high-performance ionic piezocapacitive sensors were realized. Under the synergistic effect of the low-stiffness porous hemisphere microstructure and the electric double layer of the ionic polymer film, the sensor based on an ionic polymer film with a 1:0.75 ratio of P(VDF-HFP):[EMIM][TFSI] not only achieves a sensitivity of up to 106.27 kPa^−1^ below 3 kPa, but also has a wide measurement range of over 400 kPa, which has obvious advantages in existing flexible piezocapacitive sensors. The rapid response time of 110 s and the good stability of 2300 cycles of the sensor further elucidate its practicality. The application of the sensor in pulse monitoring, speech recognition, and detection of multiple dynamic loads verifies its excellent sensing performance. In short, the proposed heat curing process can simultaneously form porous structures and surface microstructures on PDMS films, greatly simplifying the preparation process of porous hierarchical microstructures and providing a simple and feasible way to obtain high-performance flexible pressure sensors.

## 1. Introduction

With the continuous advancement of human health monitoring, flexible sensors that can sense physiological and environmental signals such as pressure [1,2], humidity [3,4], sweat [5,6], pH [7,8], and temperature [9,10] have received a lot of attention from researchers [11,12]. Flexible pressure sensors have great application prospects in wearable electronics because pressure is the most common physiological signal. Generally, flexible pressure sensors convert pressure signals into electrical signals through piezoresistive effects [13,14], piezoelectric effects [15,16], piezocapacitive effects [17,18,19], and triboelectric effects [20,21]. Among them, flexible pressure sensors based on piezocapacitive effects have the advantages of easy implementation, insensitivity to temperature and humidity, good stability, and low energy consumption. However, conventional flexible piezocapacitive sensors that rely on changes in electrode spacing to sense external pressure have low sensitivity due to their limited thickness, which limits their application range. Therefore, in recent years, researchers have introduced ionic polymer film or microstructures into flexible piezocapacitive sensors to improve their sensitivity [22,23,24,25]. For example, Cho presented an ultrasensitive ionic liquid polymer composite with a convex and wrinkled microstructure and obtained a wearable sensor with high sensitivity of 56.91 kPa^−1^ within 0–80 kPa [22]. Ma realized a piezocapacitive sensor with a micro-arrayed dielectric layer, which has a high sensitivity of 2.04 kPa^−1^ in 0–2 kPa [23]. Due to a porous pyramid dielectric layer, Yang obtained a capacitive pressure sensor with a high sensitivity of 44.5 kPa^−1^ in 0–100 Pa [24]. The combination of ionic polymer film and microstructure can further improve the sensitivity of capacitive pressure sensors. For example, based on a dielectric layer composed of arrayed cup-shaped microcolumns and ionic liquids, Li presented a capacitive pressure sensor with a high sensitivity of up to 87.75 kPa^−1^ [26]. It is worth noting that the introduction of microstructures often accompanies a decrease in the measurement range of sensors while improving their sensitivity, therefore limiting the application of the sensors. Along with this, achieving high sensitivity and wide range simultaneously has always been an important goal in the research field of flexible pressure sensors. Recently, some researchers have shown that reasonable hierarchical microstructures can ensure high sensitivity of the pressure sensors while having a wide measurement range [27,28,29]. For example, due to a PDMS flexible electrode with porous hierarchical microstructure and an ionic polymer dielectric layer, Chen realized a capacitive pressure sensor with a sensitivity of up to 92.87 kPa^−1^ and measure range of 100 kPa [27]. Therefore, the combination of porous hierarchical microstructure and ionic polymer film is expected to obtain a flexible piezocapacitive sensor with high sensitivity and a wide measurement range.

However, the development of high-performance flexible pressure sensors with porous hierarchical microstructures is limited by the complex, time-consuming, and costly preparation processes of porous hierarchical microstructures. This is because the preparation methods of inner pores are often different from the preparation methods of surface microstructures. The commonly used preparation processes for inner pores are the sacrificial template method [30], chemical porogen method [31], freeze-drying method [32], and microwave irradiation method [33]. The commonly used preparation processes for surface microstructures are the lithography [34], laser forming [35], and surface template method [36]. Therefore, porous hierarchical microstructures used for flexible sensors are typically prepared by combining multiple processes from the above processes, resulting in time-consuming and costly implementation of flexible sensors with porous hierarchical microstructures, which is not conducive to the practical application of these flexible sensors. Obviously, it is very important to develop a simple and efficient one-step preparation process for porous hierarchical microstructures, which can greatly promote the development of high-performance flexible sensors with porous hierarchical microstructures.

Herein, a modified heat curing process combining a reusable laser-assisted template with microstructures and renewable glucose monohydrate additive was first proposed to form a porous hemispherical array on a PDMS film in one step. During the PDMS heat curing process, the laser-prepared template was used to form surface microstructures on PDMS film. Meanwhile, the thermal decomposition of glucose monohydrate additive induces the formation of porous microstructures within PDMS film. Further, the obtained porous hierarchical structural PDMS films were coated with CNTs conductive layers and used as electrodes to achieve high-performance piezocapacitive sensors with ionic polymer dielectric layers. Specifically, the sensor based on a 10 × 10 porous hemispherical array and an ionic polymer film with a 1:0.75 ratio of P(VDF-HFP):[EMIM][TFSI] exhibits an ultra-high sensitivity of 106.27 kPa^−1^ below 3 kPa and a high sensitivity of 2.75 kPa^−1^ at a high pressure of 400 kPa. Meantime, the sensor also has fast response characteristics and good stability. Along with that, the sensor was used for physiological signal monitoring, dynamic load detection, and a 3 × 3 pressure sensor array, demonstrating its practical application prospects. The experimental results show that the proposed heat curing process realizes a simple, low-cost, and one-step preparation of porous hierarchical microstructures, and provides a simple and feasible approach for obtaining high-performance flexible pressure sensors with porous hierarchical microstructures.

## 2. Experimental Section

### 2.1. Materials

1-ethyl-3-methylimidazolium bis(trifluoromethylsulfonyl)imide ([EMIM][TFSI]) was obtained from Shanghai Aladdin Bio-Chem Technology Co., Ltd., Shanghai, China. Poly(vinylidene fluoride-co-hexa-fluoroproylene) (P(VDF-HFP)) copolymer was obtained from Sigma-Aldrich Inc., Milwaukee, WI, USA. Glucose monohydrate (C_6_H_12_O_6_·H_2_O) powder was obtained from Tianjin Yong Da Chemical Co., Ltd., Tianjin, China. PDMS precursor was obtained from Dow Corning Co., Ltd, Midland, MI, USA. Carbon nanotubes (CNTs) with a diameter of 20–30 nm and a length of 10–30 µm were obtained from Chengdu Organic Chemicals Co., Ltd., Chengdu, China. Acetone was obtained from Wuhan Shenshi Chemical Co., Ltd., Wuhan, China. ITO/PET electrodes were obtained from Shanghai Keyan Phosphor Technology Co., Ltd., Shanghai, China.

### 2.2. Fabrication of the Capacitive Pressure Sensor

The capacitive pressure sensor is mainly composed of the flexible PDMS/CNTs electrode with a porous hemispherical array and an ionic polymer film.

**Figure 1:**
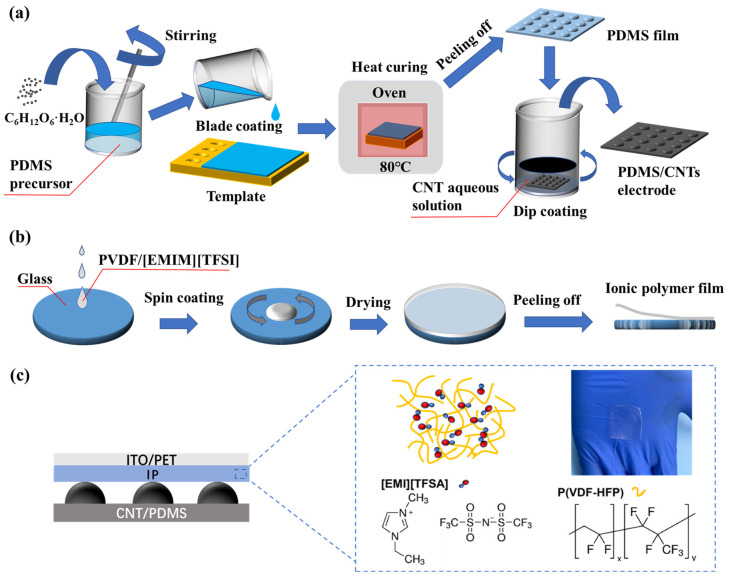
(**a**) Fabrication flow of a PDMS/CNTs electrode with a porous hemispherical array. (**b**) Fabrication flow of an ionic polymer film. (**c**) Structure diagram of the sensor, composition of ionic polymer, and physical photo of an ionic polymer film.

The fabrication flow of the flexible PDMS/CNTs electrode with a porous hemispherical array is illustrated in Figure 1a. First, a copper template with a 10 × 10 hemispherical hole array was prepared using a nanosecond laser with a pulse frequency of 20 kHz, a power of 24 W, a scanning speed of 500 mm/s, a pulse width of 10 ns, and a wavelength of 1064 nm. Then, the PDMS precursor was mixed with the glucose monohydrate powder in a 3:1 mass ratio and stirred for 1 h to form a glucose/PDMS mixture. The mixture was uniformly scraped onto the copper template and cured by heating in an oven at 80 °C for 2 h to form a 0.5 mm thick PDMS film with a porous hemispherical structure. Then, the PDMS film with a porous hemispherical array was peeled off from the template and immersed in an aqueous solution containing 3% wt CNTs. Subsequently, the aqueous solution was stirred for 12 h, and CNTs in the solution were coated on the PDMS surface to form a conductive layer. Finally, the PDMS film with CNTs conductive layer was heated in a 50 °C oven for 1 h to increase the adhesion between CNTs and PDMS, thus achieving a flexible PDMS/CNTs electrode with a porous hemispherical array.

The fabrication flow of the ionic polymer film is illustrated in Figure 1b. First, P(VDF-HFP) copolymer was mixed with acetone in a 7:1 mass ratio and stirred for 8 h to obtain a P(VDF-HFP) dissolved solution. Then, the solution was mixed with [EMIM][TFSI] at mass ratios of 1:0.25, 1:0.5, and 1:0.75, respectively, to achieve three ratios of ionic polymer precursors. At last, the ionic polymer precursors were spin-coated on a glass plate to obtain ionic polymer films with a thickness of about 200 μm.

Finally, the PDMS/CNTs electrode with a porous hemispherical array, the ionic polymer (IP) film, and a polyethylene terephthalate (PET) film with ITO conductive layer were assembled sequentially to obtain a capacitive pressure sensor, as shown in Figure 1c. The ionic polymer film, as the dielectric layer, is responsible for providing mobile ions to form an electric double layer (EDL) under the action of voltage and contact pressure, which consists of [EMI][TFSA] and P(VDF-HFP). The optical photograph in the picture shows that the ionic polymer film is a transparent soft film.

Meanwhile, to study the effect of porous microstructure on sensor performance, a capacitive pressure sensor with a hemispherical array was prepared through the above process without the addition of glucose monohydrate powder.

### 2.3. Characterization and Measurement

The morphological characteristics of samples were observed using a scanning electron microscopy (SEM, Carl Zeiss AG Co., Ltd., Oberkochen, Germany). The mechanical and electrical response characteristics of sensors were measured synergistically using a general mechanical tester (BAB-50MT, Transcell Electronic Co., Ltd., Chicago, IL, USA) and a capacitance meter (ET4401, Hangzhou ZhongChuang Co., Ltd., Hangzhou, China). During the testing process, the general mechanical tester provides external pressure to act on the sensor, and records the external pressure and strain data of the sensor in real time. At the same time, the capacitance meter records in real-time the capacitance changes of the sensor under external pressure.

### 2.4. Calculations

The key parameter sensitivity (***S***) of piezocapacitive sensor can be obtained by the following equation:

(1) $$S=\frac{\Delta C/C_{0}}{\Delta P}$$

where ***C*_0_** denotes the initial capacitance of the sensor, Δ***P*** denotes the change value of the external pressure loaded on the sensor, and Δ***C*** denotes the change value in capacitance of the sensor corresponding to the change value Δ***P*** of external pressure.

## 3. Results and Discussion

### 3.1. Formation Mechanism of Porous Hemispherical Structure on PDMS

**Figure 2:**
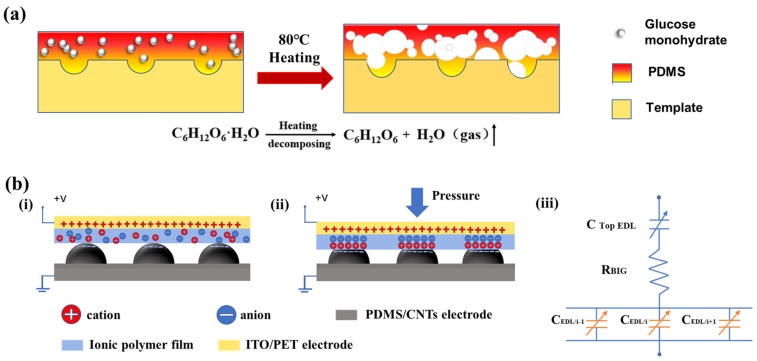
(**a**) Formation mechanism of porous hierarchical structure on PDMS substrate. (**b**) Working mechanism diagram of the sensor with an ionic polymer film.

The formation mechanism of porous hemispherical structure on PDMS film is illustrated in Figure 2a. When the mixture of PDMS precursor and glucose monohydrate scraped onto a copper template is heated at 80 °C, the PDMS precursor in the mixture is thermally solidified into a PDMS film, and thus the hemispherical array on the template is transferred to the surface of the PDMS film. Meanwhile, the water molecule of glucose monohydrate in the mixture is decomposed into gaseous water during heating, and the diffusion of gaseous water in the PDMS precursor leads to the formation of porous structure inside the PDMS film. Therefore, porous PDMS film with a porous hemispherical structure is achieved through a simple and low-cost heat curing process in one step.

### 3.2. Sensing Mechanism of Sensor

The sensing mechanism of the piezocapacitive sensor is shown in Figure 2b. When external pressure is not applied on the sensor, the ionic polymer film is not in close contact with the electrodes, so the anions and cations in the ionic polymer film are randomly distributed, as shown in Figure 2b(i). When the sensor is compressed, the ionic polymer film is in close contact with the electrodes. Under the action of external pressure and an electric field, the anions in the ionic polymer film gather to the contact zone between the film and the positive electrode, and the cations in the ionic polymer film gather to the contact zone between the film and the negative electrode, leading to the occurrence of an EDL, as shown in Figure 2b(ii). The EDL leads to the formation of two new interface capacitances between the upper and lower electrodes in the piezocapacitive sensor. Figure 2b(iii) illustrates the equivalent circuit model of the sensor. In the model, ***C*_TopEDL_** denotes the interface capacitance between the ionic polymer film and the upper electrode, ***C*_EDL/i_** denotes the interface capacitance between the ionic polymer film and the hemispheres on the bottom PDMS-based electrode, and ***R*_BIG_** denotes the internal resistance of the ionic polymer film. According to the model, the value of total capacitance ***C*_total_** of the sensor can be obtained by the following equation:

(2) $$C_{\mathrm{total}}=\frac{1}{\frac{1}{\sum_{i=1}^{n}C_{\mathrm{EDL}/i}}+\frac{1}{C_{\mathrm{TopEDL}}}}$$

The model shows that the sensing performance of the piezocapacitive sensor mainly depends on the interface capacitances induced by EDL, which is affected not only by the distance between the upper and lower electrodes but also by the contact area between the electrodes and the ionic polymer film. Obviously, the change rate of interface capacitance generated by EDL is greater than that of the traditional capacitance determined by electrode spacing. Therefore, the combination of ionic polymer dielectric layer and porous hemispherical electrode is expected to obtain a highly sensitive piezocapacitive sensor.

### 3.3. Sample Morphology

**Figure 3:**
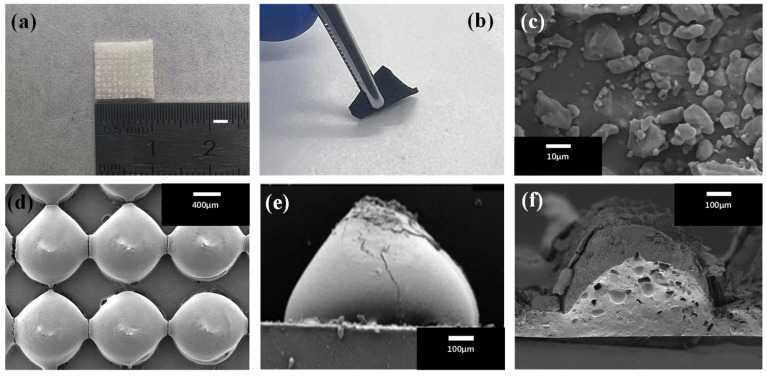
Photos of (**a**) a PDMS substrate, and (**b**) a PDMS/CNTs electrode with a porous hemispherical array. SEM photos of (**c**) glucose monohydrate microparticles, (**d**) a porous hemispherical array, (**e**) side view of a hemisphere covered with CNTs, and (**f**) cross-section of a porous hemisphere.

The morphologies of samples were observed, as shown in Figure 3. The optical photo in Figure 3a displays a 10 mm × 10 mm × 0.5 mm PDMS substrate with a 10 × 10 porous hemispherical array. After being immersed in CNTs suspension, the color of the PDMS substrate completely changed from white to black, as shown in Figure 3b, indicating that CNTs are effectively attached to the PDMS surface and PDMS/CNTs flexible electrode is obtained. Figure 3c shows monohydrate glucose microparticles with a size of about 10 μm, which is added to the PDMS precursor as pore-forming agents. The SEM photo in Figure 3d shows that the hemispheres covered with CNT’s conductive layer are neatly arranged on the PDMS/CNTs electrode to form a hemispherical array, and the dimensions of the hemispheres are basically the same. The SEM photo in Figure 3e shows a side view of a hemisphere covered with CNTs, which has a radius of 400 μm. Figure 3f shows a cross-sectional view of a porous hemisphere on PDMS/CNTs electrode. In the figure, there are some pores larger than 50 μm in size, which are larger than glucose monohydrate microparticles, confirming the pore-forming effect of glucose monohydrate microparticles.

### 3.4. Mechanical Simulation of PDMS Film with Hemispherical Array

**Figure 4:**
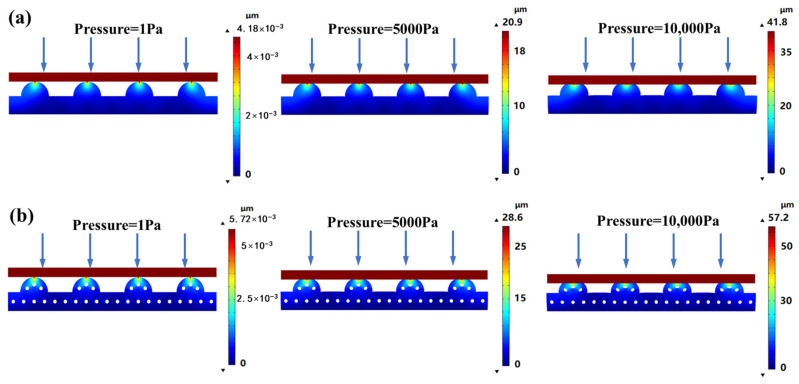
FEA simulation results of the PDMS substrates with (**a**) a solid hemispherical array, and (**b**) a porous hemispherical array at 1 Pa, 5000 Pa, and 10,000 Pa pressures.

To theoretically study the influence of porous hemispherical structure on the performance of PDMS-based pressure sensor, the pressure–deformation relationship of PDMS substrates with hemispherical array were simulated by the COMSOL Multiphysics 6.0 simulator (COMSOL Inc., Stockholm, Sweden). In the simulation model, the radius of the hemisphere on the PDMS substrate is 400 μm, and the size of the pores inside the PDMS is 50 μm. The density, elastic modulus, and Poisson’s ratio of the substrate are selected as 0.970 g/cm^3^, 500 kPa, and 0.49, respectively. The simulation model is divided into finite elements using a free triangular mesh. During the simulation process, the bottom of the model is fixed, and pressure is uniformly applied to the top of the model. The finite element analysis (FEA) results are illustrated in Figure 4. In the figure, at 1 Pa pressure, the deformation of PDMS substrate with porous hemispherical array is 5.72 nm, which is higher than 4.18 nm of PDMS substrate with solid hemispherical array. At 5000 Pa pressure, the deformation of PDMS substrate with porous hemispherical array is 28.6 μm, which is higher than 20.9 μm of PDMS substrate with solid hemispherical array. At 10,000 Pa pressure, the deformation of PDMS substrate with porous hemispherical array is 57.2 μm, which is higher than 41.8 μm of PDMS substrate with solid hemispherical array. Obviously, the porous structure allows the sensor to have greater deformation, which is beneficial for improving the sensitivity of the sensor.

### 3.5. Sensing Characterization of the Sensor

**Figure 5:**
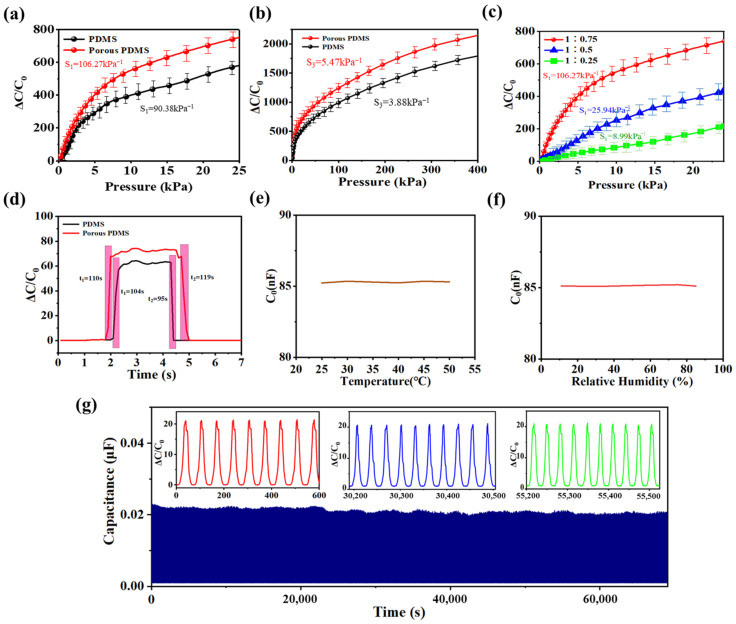
Sensitivities of the sensors with (**a**,**b**) solid hemispherical array PDMS, porous hemispherical array PDMS, and ionic polymer film with a ratio of 1:0.75 (P(VDF-HFP):[EMIM][TFSI]), and (**c**) different ratios of ionic polymer films. (**d**) Response time test, (**e**) temperature test, (**f**) relative humidity test, and (**g**) cycle stability test of the sensor.

The sensing characteristics of the sensors were measured, as shown in Figure 5. Figure 5a shows the measured sensitivities of the capacitive pressure sensors based on PDMS/CNTs electrodes with hemisphere array and an ionic polymer film with a 1:0.75 ratio of P(VDF-HFP):[EMIM][TFSI] under external pressure. In the figure, the sensitivity of the sensor decreases with increasing pressure. This is because when the sensor begins to be subjected to external pressure, the porous hemispherical microstructure on the upper layer of the PDMS film first undergoes deformation, rapidly causing a change in the sensor capacitance. As the external pressure increases, the deformation of the sensor mainly comes from the compression of the porous structure below the PDMS film. Under the same pressure, the deformation of the lower porous structure of the PDMS film is smaller than that of the upper porous hemispherical microstructure of the PDMS film, resulting in a decrease in the sensitivity of the sensor. The sensor with porous hemisphere array exhibits an ultrahigh sensitivity of 106.27 kPa^−1^ in the 0–3 kPa pressure range, which is higher than 90.38 kPa^−1^ of the sensor with solid hemisphere array. This is because the porous structure reduces the stiffness of the PDMS substrate, making the sensor more prone to deformation under external pressure, which has been verified by the above theoretical simulation. The deformation of the sensor is proportional to the change in contact area between the ion polymer film and the electrode. A large change in contact area leads to a large change in interface capacitance of the sensor, so the sensor with greater deformation has higher sensitivity. The measured result in Figure 5b show that the sensor still has a high sensitivity of 2.75 kPa^−1^ at 400 kPa, indicating that the sensor has achieved both a wide measurement range and high sensitivity. This can be attributed to the reasonable size of the hemisphere. Along with this, the proportion of P(VDF-HFP):[EMIM][TFSI] of ionic polymer dielectric layer also affects the sensing performance of the sensor, as shown in Figure 5c. It can be seen from the figure that the sensitivity of the corresponding sensor increases with the increase of the proportion of [EMIM] [TFSI] in the ionic polymer dielectric layer, which is attributed to the more mobile ions brought by the higher proportion of [EMIM] [TFI]. Under the same pressure, the more mobile ions of ion gel dielectric layer, the greater the change in the interfacial capacitance of the EDL in the sensor, resulting in a higher sensitivity. Therefore, among these samples, the sensor based on an ionic polymer film with a 1:0.75 ratio of P(VDF-HFP):[EMIM][TFSI] has the highest sensitivity. Unless otherwise stated, the sensor based on an ionic polymer film with a 1:0.75 ratio of P(VDF-HFP):[EMIM][TFSI] was used for subsequent testing and applications. Table 1 lists a comparison of the comprehensive performance of the sensor with some recently reported flexible piezocapacitance sensors. The sensitivity and measurement range of the sensor prepared by the modified heat curing process are significantly better than those of other sensors.

Stability and fast responses are important for the practical application of pressure sensors. Figure 5d shows the response curve of the sensors under an instantaneous loading-unloading pressure of 500 Pa. It can be seen from the figure that the response times and recovery times of the sensor with porous hemispherical array under 500 Pa pressure are 110 ms and 119 ms, respectively, indicating that the sensor is suitable for fast response situations. The response speed of the sensor with a solid hemispherical structure is slightly faster than that of the sensor with porous hemispherical structure because the viscoelasticity of the sensor with a solid hemispherical structure is lower than that of the sensor with a porous hemispherical structure. Figure 5e,f show the effects of temperature and relative humidity on the electrical performance of the sensor at a pressure of 5 kPa, respectively. As can be seen from the figures, the capacitance of the sensor does not change with temperature and relative humidity, indicating the advantages of the capacitance sensor. Besides, a 1000 Pa pressure was continuously loaded and unloaded onto the sensor for 2300 cycles to test its stability. The corresponding response curve of the sensor is shown in Figure 5g. During stability testing process, the amplitude of the response signal of the sensor to the 1000 Pa pressure is basically unchanged. Meanwhile, the three illustrations in the figure show that the response signals of the sensor at the beginning, middle, and end stages of the cycle are basically consistent, further proving that the sensor has good stability.

### 3.6. Application of the Sensor

**Figure 6:**
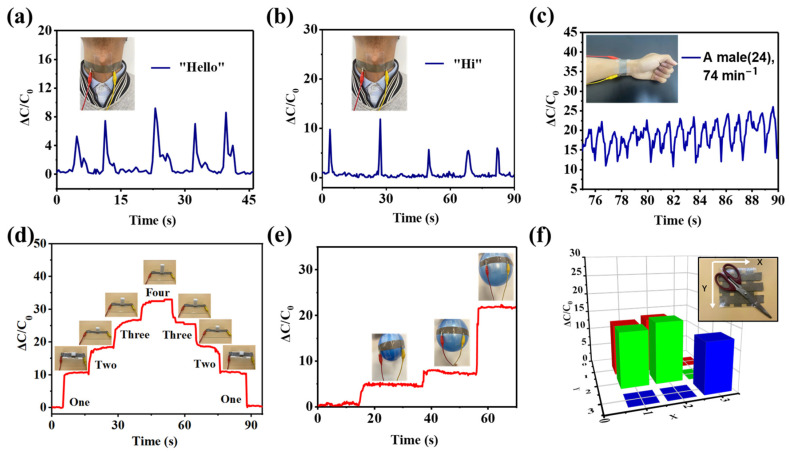
Application of the sensor in (**a**,**b**) speech recognition, (**c**) artery pulse monitoring, (**d**) sugar particle weighing tests, and (**e**) balloon expansion detection. (**f**) A 3 × 3 sensing array for detecting a scissor.

The sensor was used to monitor several physiological signals to evaluate its application in health monitoring. As shown in Figure 6a,b, the sensor was attached to the throat of volunteers to detect throat vibrations during speech. When the volunteer said the two-syllable word “Hello,” the sensor clearly responded to two successive peak electrical signals. When volunteers said the monosyllabic word ‘Hi,’ the sensor only output one peak electrical signal. Moreover, the higher the tone of speech, the higher the peak of the electrical signal responded by the sensor. The experiment results indicate that the sensor has potential applications in the field of sound signal collection. In Figure 6c, the sensor was positioned at the radial artery of the volunteer to detect the pulse signal, and the sensor gave a periodic electrical signal of approximately 74 beats per minute, demonstrating the high sensitivity of the sensor. Figure 6d shows a trapezoidal electrical signal response curve of the sensor caused by dynamic load. In the figure, four pieces of sugar (each with a mass of about 1 g) were placed on or removed from the sensor in sequence, and the response signal of the sensor also underwent similar amplitude changes in sequence, demonstrating the stability of the sensor. In Figure 6e, the sensor was attached to the surface of a balloon to detect changes in balloon volume. The larger the balloon volume, the greater the response electrical signal output by the sensor. It indicates that the sensor has high sensitivity and stable sensing performance. Along with this, a 3 × 3 array of pressure sensors was fabricated to collect mechanical signals in a 2D planar region. In Figure 6f, a scissor was placed on the sensing array, and the relative capacitance changes in the nine regions matched the distribution of pressure applied by the scissor, indicating the potential application of the sensor in electronic skin.

## 4. Conclusions

In summary, with the help of a laser-assisted template and glucose monohydrate additive, a modified heat curing process was used for the first time to achieve one-step preparation of porous PDMS substrates with a hemispherical array. Subsequently, the porous PDMS substrates coated with CNT conductive layers were applied to capacitive pressure sensors with ionic polymer dielectric layers. Due to the excellent flexibility of the porous hemispherical microstructure and the electric double layer in the ionic polymer dielectric layer, the sensor based on an ionic polymer film with a 1:0.75 ratio of P(VDF-HFP):[EMIM][TFSI] has an ultra-high sensitivity of 106.27 kPa^−1^ below 3 kPa and a high sensitivity of 2.75 kPa^−1^ at a high pressure of 400 kPa, which has obvious advantages in existing flexible piezocapacitive sensors. Meanwhile, the sensor has a quick response of 119 ms and a good stability over 2300 cycles. The application of the sensor in pulse monitoring, speech recognition, and detection of multiple dynamic loads further demonstrates its practical application prospects. The experimental results show that the modified heat curing process provides a simple and feasible way to obtain flexible pressure sensors with high sensitivity and wide measurement range. Especially, the new modified heat curing process realizes the one-step preparation of porous hierarchical microstructures, which effectively simplifies the existing preparation process of porous hierarchical microstructures and is expected to promote the application of porous hierarchical microstructures in the fields of flexible electronics, biological monitoring, and water treatment.

## Figures and Tables

**Table 1:** Comprehensive performance comparison of the sensors.

Electrode	Dielectric Layer	Sensitivity	Sensing Range	Response/Recovery Time	Ref.
aluminum foil	PVA/KOH/KI/glycerol	0.3199	0.1 kPa–65 kPa	80 ms/55 ms	[2]
AgNWs/PDMS	PDMS	2.04 kPa^−1^	0 Pa–9 kPa	100 ms/100 ms	[23]
ITO/PET	MWCNT/PDMS	1.448 Pa^−1^	0 Pa–20 kPa	123 ms/86 ms	[25]
AgNWs/PDMS	[EMIM][TFSI]/PVDF-HFP	37.8 kPa^−1^	24 Pa–90 kPa	78 ms/47 ms	[29]
Al Fabric	[EMIM][TFSI]/ PVDF-HFP/PDMS	56.91 kPa^−1^	0 kPa–80 kPa	60 ms/50 ms	[30]
MXene/ nonwoven fabric	[EMIM][TFSI]/PVDF-HFP	31.40 kPa^−1^	0 kPa–80 kPa	45 ms/45 ms	[32]
CNTs/PDMS	[EMIM][TFSI]/PVDF-HFP	106.27 kPa^−1^	0 kPa–400 kPa	110 ms/119 ms	This work

## Data Availability

No new data were created or analyzed in this study. Data sharing is not applicable to this article.

## References

[1] Xu C., Mei D., Zhu L., Wang Y. (2022). Flexible capacitive pressure sensor array using acoustic-assisted fabrication of microstructures as surface and dielectric layers. Sens. Actuators A Phys..

[2] Zeng Y., Qin Y., Yang Y., Lu X. (2022). A low-cost flexible capacitive pressure sensor for health detection. IEEE Sens. J..

[3] Duan Z., Yuan Z., Jiang Y., Zhao Q., Huang Q., Zhang Y., Liu B., Tai H. (2022). Power generation humidity sensor based on primary battery structure. Chem. Eng. J..

[4] Zhu P., Kuang Y., Wei Y., Li F., Ou H., Jiang F., Chen G. (2021). Electrostatic self-assembly enabled flexible paper-based humidity sensor with high sensitivity and superior durability. Chem. Eng. J..

[5] Liang B., Cao Q., Mao X., Pan W., Tu T., Fang L., Ye X. (2021). An integrated paper-based microfluidic device for real-time sweat potassium monitoring. IEEE Sens. J..

[6] Li M., Wang L., Liu R., Li J., Zhang Q., Shi G., Li Y., Hou C., Wang H. (2021). A highly integrated sensing paper for wearable electrochemical sweat analysis. Biosens. Bioelectron..

[7] Tang Y., Zhong L., Wang W., He Y., Han T., Xu L., Mo X., Liu Z., Ma Y., Bao Y. (2022). Recent advances in wearable potentiometric Ph sensors. Membranes.

[8] Mazzara F., Patella B., D’Agostino C., Bruno M.G., Carbone S., Lopresti F., Aiello G., Torino C., Vilasi A., O’Riordan A. (2021). Pani-based wearable electrochemical sensor for Ph sweat monitoring. Chemosensors.

[9] Chen L., Chang X., Wang H., Chen J., Zhu Y. (2022). Stretchable and transparent multimodal electronic-skin sensors in detecting strain, temperature, and humidity. Nano Energy.

[10] Wu Z., Ding H., Tao K., Wei Y., Gui X., Shi W., Xie X., Wu J. (2021). Ultrasensitive, stretchable, and fast-response temperature sensors based on hydrogel films for wearable applications. ACS Appl. Mater. Interfaces.

[11] Hong Y.J., Jeong H., Cho K.W., Lu N., Kim D. (2019). Wearable and implantable devices for cardiovascular healthcare: From monitoring to therapy based on flexible and stretchable electronics. Adv. Funct. Mater..

[12] Liu X., Miao J., Fan Q., Zhang W., Zuo X., Tian M., Zhu S., Zhang X., Qu L. (2022). Recent progress on smart fiber and textile based wearable strain sensors: Materials, fabrications and applications. Adv. Fiber Mater..

[13] Zhu J., Xue X., Song Y., Wu C., Mao Q. (2022). A flexible and robust double-helix tubular cloth/carbon fibers piezoresistive sensor for human motion detecting. Mater. Lett..

[14] Lei X., Ma L., Yu S., Ren T., Li S., Yuan J., Liu F. (2023). Ultrahigh sensitive, sensing-actuating integrated, and multi-functional intelligent skin based on electromechanical-hydraulic coupling. Chem. Eng. J..

[15] Yu J., Xian S., Zhang Z., Hou X., He J., Mu J., Geng W., Qiao X., Zhang L., Chou X. (2023). Synergistic piezoelectricity enhanced BaTiO_3_/Polyacrylonitrile elastomer-based highly sensitive pressure sensor for intelligent sensing and posture recognition applications. Nano Res..

[16] Liu G., Chen X., Li X., Wang C., Tian H., Chen X., Nie B., Shao J. (2022). Flexible, equipment-wearable piezoelectric sensor with piezoelectricity calibration enabled by in-situ temperature self-sensing. IEEE Trans. Ind. Electron..

[17] Mishra R.B., El-Atab N., Hussain A.M., Hussain M.M. (2021). Recent progress on flexible capacitive pressure sensors: From design and materials to applications. Adv. Mater. Technol..

[18] Zhang S., Wang L., Luo Y., Wang K., Feng X., Pei Y., Wu H., Li Y., Wang Z., Lu B. (2023). A convenient, low-cost graphene UV-cured additive manufacturing electronic process to achieve flexible sensors. Chem. Eng. J..

[19] Zammali M., Liu S., Yu W. (2022). A Flexible, transparent, ultralow detection limit capacitive pressure sensor. Adv. Mater. Interfaces.

[20] Zhong Y., Wang J., Han L., Dai S., Zhu H., Hua J., Cheng G., Ding J. (2023). High-performance flexible self-powered triboelectric pressure sensor based on chemically modified micropatterned PDMS film. Sens. Actuators A Phys..

[21] Zhao L., Zou H., Wei K., Zhou S., Meng G., Zhang W. (2023). Mechanical Intelligent Energy Harvesting: From Methodology to Applications. Adv. Energy Mater..

[22] Cho C., Kim D., Lee C., Oh J.H. (2023). Ultrasensitive ionic liquid polymer composites with a convex and wrinkled microstructure and their application as wearable pressure sensors. ACS Appl. Mater. Interfaces.

[23] Ma L., Shuai X., Hu Y., Liang X., Zhu P., Sun R., Wong C. (2018). A highly sensitive and flexible capacitive pressure sensor based on a micro-arrayed polydimethylsiloxane dielectric layer. J. Mater. Chem. C.

[24] Yang J.C., Kim J.O., Oh J., Kwon S.Y., Sim J.Y., Kim D.W., Choi H.B., Park S. (2019). Microstructured porous pyramid-based ultrahigh sensitive pressure sensor insensitive to strain and temperature. ACS Appl. Mater. Interfaces.

[25] Lv C., Tian C., Jiang J., Dang Y., Liu Y., Duan X., Xie M. (2023). Ultrasensitive linear capacitive pressure sensor with wrinkled microstructures for tactile perception. Adv. Sci..

[26] Li L., Zhu G., Wang J., Chen J., Zhao G., Zhu Y. (2023). A flexible and ultrasensitive interfacial iontronic multisensory sensor with an array of unique “cup-shaped” microcolumns for detecting pressure and temperature. Nano Energy.

[27] Chen H., Guo D., Lei X., Wu W., Guo X., Li Y., Liu F. (2023). One-step laser direct-printing process of a hybrid microstructure for highly sensitive flexible piezocapacitive sensors. ACS Appl. Mater. Interfaces.

[28] Chen J., Li L., Zhu Z., Luo Z., Tang W., Wang L., Li H. (2022). Bioinspired design of highly sensitive flexible tactile sensors for wearable healthcare monitoring. Mater. Today Chem..

[29] Xu J., Li H., Yin Y., Li X., Cao J., Feng H., Bao W., Tan H., Xiao F., Zhu G. (2022). High sensitivity and broad linearity range pressure sensor based on hierarchical in-situ filling porous structure. NPJ Flexble Electron..

[30] Zhao T., Li T., Chen L., Yuan L., Li X., Zhang J. (2019). Highly sensitive flexible piezoresistive pressure sensor developed using biomimetically textured porous materials. ACS Appl. Mater. Interfaces.

[31] Long Y., Zhao X., Jiang X., Zhang L., Zhang H., Liu Y., Zhu H. (2018). A porous graphene/polydimethylsiloxane composite by chemical foaming for simultaneous tensile and compressive strain sensing. FlatChem.

[32] Huang W., Dai K., Zhai Y., Liu H., Zhan P., Gao J., Shen C. (2017). Flexible and lightweight pressure sensor based on carbon nanotube/thermoplastic polyurethane-aligned conductive foam with superior compressibility and stability. ACS Appl. Mater. Interfaces.

[33] Zhao Y., Lei X., Xu J., Li Y., Wu W., Guo X., Liu F. (2022). High-performance porous PDMS-based piezoresistive sensor prepared by a modified microwave irradiation process. ACS Appl. Electron. Mater..

[34] Luo Y., Chen X., Tian H., Li X., Lu Y., Liu Y., Shao J. (2022). Gecko-inspired slant hierarchical microstructure-based ultrasensitive iontronic pressure sensor for intelligent interaction. Research.

[35] Zhang C., Zhou W., Geng D., Bai C., Li W., Chen S., Xie Y. (2021). Laser direct writing and characterizations of flexible piezoresistive sensors with microstructures. Opto-Electron. Adv..

[36] Guo X., Ma L., Wu W., Li S., Lei X., Wu X., Liu F. (2022). Ultra-sensitive flexible piezoresistive pressure sensor prepared by laser-assisted copper template for health monitoring. Sens. Actuators A Phys..

